# Soluble RAGE Treatment Delays Progression of Amyotrophic Lateral Sclerosis in SOD1 Mice

**DOI:** 10.3389/fncel.2016.00117

**Published:** 2016-05-09

**Authors:** Judyta K. Juranek, Gurdip K. Daffu, Matthew S. Geddis, Huilin Li, Rosa Rosario, Benjamin J. Kaplan, Lauren Kelly, Ann Marie Schmidt

**Affiliations:** ^1^Division of Endocrinology, Department of Medicine, New York University Langone Medical CenterNew York, NY, USA; ^2^Department of Surgery, Columbia University Medical CenterNew York, NY, USA; ^3^Department of Science, Borough of Manhattan Community College-City University of New YorkNew York, NY, USA; ^4^Division of Biostatistics, Department of Population Health, New York University Langone Medical CenterNew York, NY, USA

**Keywords:** amyotrophic lateral sclerosis, SOD1, spinal cord, RAGE, soluble RAGE, motor neurons

## Abstract

The etiology of amyotrophic lateral sclerosis (ALS), a fatal motor neuron disorder characterized by progressive muscle weakness and spasticity, remains largely unknown. Approximately 5–10% of cases are familial, and of those, 15–20% are associated with mutations in the gene encoding Cu/Zn superoxide dismutase (SOD1). Mutations of the SOD1 gene interrupt cellular homeostasis and contribute to cellular toxicity evoked by the presence of altered SOD1, along with other toxic species, such as advanced glycation end products (AGEs). AGEs trigger activation of their chief cell surface receptor, RAGE (receptor for advanced glycation end products), and induce RAGE-dependent cellular stress and inflammation in neurons, thereby affecting their function and leading to apoptosis. Here, we show for the first time that the expression of RAGE is higher in the SOD1 transgenic mouse model of ALS vs. wild-type mouse spinal cord. We tested whether pharmacological blockade of RAGE may delay the onset and progression of disease in this mouse model. Our findings reveal that treatment of SOD1 transgenic mice with soluble RAGE (sRAGE), a natural competitor of RAGE that sequesters RAGE ligands and blocks their interaction with cell surface RAGE, significantly delays the progression of ALS and prolongs life span compared to vehicle treatment. We demonstrate that in sRAGE-treated SOD1 transgenic animals at the final stage of the disease, a significantly higher number of neurons and lower number of astrocytes is detectable in the spinal cord. We conclude that RAGE antagonism may provide a novel therapeutic strategy for ALS intervention.

## Introduction

Amyotrophic lateral sclerosis (ALS), also known as Lou Gehrig's disease, is a progressive and terminal neurological disorder, which results in severe disability and death within 5 years after diagnosis (Ferraiuolo et al., 2011). Approximately 5–10% of cases are familial, and of those, 15–20% are associated with mutations in the gene encoding Cu/Zn superoxide dismutase (SOD1; Andersen and Al-Chalabi, 2011). Multiple contributory factors in the pathogenesis of ALS include enhanced oxidative stress, neuroinflammation, glutamate toxicity, mitochondrial dysfunction, neurofilament disorganization, disordered axonal transport, and neuronal apoptosis (Ferraiuolo et al., 2011). Mutations of the SOD1 gene not only lead to the loss of biological function of the SOD1 molecule but they also disrupt normal cellular homeostasis and result in accumulation and aggregation of mutated SOD1 as well as other molecular species such as advanced glycation end products (AGEs; Albers and Beal, 2000; Kato et al., 2001). AGEs trigger activation of RAGE (receptor for advanced glycation end products) and along with reactive oxygen species are primary contributors to cellular stress, endoplasmic reticulum stress, accumulation of abnormal protein aggregates, and mitochondrial dysfunction (Daffu et al., 2013).

RAGE, a member of immunoglobulin superfamily and a multiligand cell surface receptor, binds to AGEs, such as carboxymethyllysine (CML), and interacts with a number of other molecules such as S100/calgranulin family members, High Mobility Group Box-1 (HMGB1), and molecules that are prone to aggregation and post translational modifications (Ding and Keller, 2005), such as amyloid-β-peptide, linked to Alzheimer's disease (AD) and neurodegeneration (Chen et al., 2007; Schmidt et al., 2009). Recently, we demonstrated that RAGE and its inflammatory ligands S100B, CML-AGE, and HMGB1, were co-expressed and upregulated in thoracic spinal cord samples of human ALS patients compared to controls (Juranek et al., 2015).

Soluble RAGE (sRAGE) is a soluble form of RAGE lacking the transmembrane region, composed of the extracellular domains of the receptor. sRAGE acts as a RAGE decoy, thereby inhibiting RAGE function and lowering levels of RAGE ligands, effectively preventing harmful RAGE ligand effects on cells (Schmidt, 2015). sRAGE can be produced and purified *in vitro* from a baculovirus expression system, consisting of the three extracellular RAGE domains, V-C-C, and can be used as a biological agent (Schmidt et al., 1999). In pre-clinical studies in animal models, therapeutic administration of sRAGE has been shown to be beneficial in several conditions such as improving wound healing in diabetic mice (Goova et al., 2001), protection of the liver from reperfusion injury (Zeng et al., 2004), suppression of periodontal bone loss in diabetic mice with infection-mediated periodontitis (Lalla et al., 2000), prevention of transmission of post-transplantation autoimmune diabetes in NOD/scid mice (Chen et al., 2004), and marked reduction in experimental autoimmune encephalomyelitis (EAE) symptoms in the mouse model of multiple sclerosis (MS; Yan et al., 2003). Other studies have shown that sRAGE treatment was beneficial in diabetic atherosclerosis, polycystic kidney disease, and autoimmune myocarditis, alleviating symptoms, and improving overall outcome of the disease (Jandeleit-Dahm et al., 2005; Yang et al., 2014; Lee et al., 2015). Furthermore, in acute liver damage secondary to massive hepatectomy, administration of sRAGE prevented excess mortality (Cataldegirmen et al., 2005).

In the present study, we tested for the first time whether sRAGE treatment offers protection against premature cell death in motor neurons of SOD1 transgenic mouse, thereby delaying the onset and progression of ALS and improving overall health status of these mice. Our findings demonstrate the upregulation of RAGE in the spinal cord of SOD1 mice, and show that sRAGE-treated SOD1 mice display improved lifespan, improved functional motor performance scores, and less neuronal cell death compared to vehicle-treated age-matched SOD1 transgenic mice controls. The results of this first study testing RAGE as a therapeutic target in ALS support further examination of this receptor in this incurable disorder.

## Materials and methods

### Animals

Eight week old transgenic B6SJL-Tg (SOD1^*^G93A) 1Gur/J mice of both genders (stock number 0002726; Jackson Laboratories, Bar Harbor, ME, USA) were used in the study. According to the Jackson Laboratories site (http://jaxmice.jax.org/strain/002726.html), transgenic mice have an abbreviated life span: 50% survive at 128.9 ± 9.1 days (Dal Canto and Gurney, 1994; Alexander et al., 2004). These numbers were consistent with our experimental records. Transgenic SOD1 females were kept as internal controls and all males were weight-matched and randomly divided into two groups; mice were injected daily with either vehicle (murine serum albumin, MSA, 175 μg/day IP; Sigma Aldrich, St. Louis MO, USA) or soluble RAGE (175 μg/day IP; Park et al., 1998) beginning at 8 weeks of age and continued until sacrifice at the terminal stage of the disease defined as the end of the experiment. The final stage of the disease, as described by the institutional guidelines issued by local Animal Care and Use Committees of Columbia and New York Universities, was determined by 20% weight loss or the animal's inability to right itself within 20 s when placed on its side (Lautenschlager et al., 2013; Parone et al., 2013). The disease onset, accompanied by posture and gait impairment, was defined as the time when animals lost ~10% of their maximal weight, before any noticeable decrease in motor performance tests (Parone et al., 2013). All experiments were approved by Institutional Animal Care and Use Committees of Columbia and New York Universities. All animals were housed and treated in accordance with ethical committee guidelines outlined in the Principles of Laboratory Animal Care, NIH publication no. 86–23, with further revisions). As there is a protective effect of estrogen and other gender-related factors on the onset and progression of the disease in females, to assure consistency of results, only male mice were used for sRAGE studies (lifespan assessment and functional performance experiments; Veldink et al., 2003; Heiman-Patterson et al., 2005).

### RAGE immunohistochemistry

Prior to the sRAGE injection experiment, a small group of control and SOD1 mice were set aside to first investigate the expression of RAGE in the lumbar spinal cord. Control and SOD1 transgenic mice were sacrificed at the same time that corresponded to the end stage of the disease as described earlier (Juranek et al., 2013a). Briefly, immediately after collection, samples were transferred to fixative (4% paraformaldehyde in distilled water) for 12 h, cryoprotected in 20% sucrose solution for 24 h, mounted in optimal cutting temperature compound (Tissue-Tek Optimal Cutting Solution, Sakura Finetek, Zoeterwoude, Netherlands) and stored at −20°C for further processing. Frozen samples were cut transversely at 10 μm thickness on cryostat (Microm HM 550, ThermoFisher Scientific, Waltham, MA, USA) and collected on polylysine-coated slides (SuperFrost Plus, Fisher Scientific, Pittsburgh, PA, USA), always in the same order—two control and two SOD1 sections from one pair of animals per one slide set. After slide collection, sections were allowed to dry for 2 h at room temperature and processed according to standard staining protocol. For immunofluorescence, dried sections were incubated with blocking solution (Cas-block, Invitrogen, Carlsbad, CA, USA) for 1 h and incubated overnight with primary goat anti-RAGE IgG (1:100; Genetex, Irvine, CA, USA). The following day sections were rinsed 4 × 5 min in phosphate buffer saline (PBS), incubated with goat anti-rabbit Alexa 594 (1:300; ThermoFisher Scientific, Waltham, MA, USA) for 1 h, rinsed again 4 × 5 min in PBS and mounted in Vectashield fluorescent mounting medium with a blue fluorescent dye DAPI (Vector Laboratories, Burlingame, CA, USA). To control specificity of secondary antibodies and minimize risk of false positive results, standard immunostaining procedure with omission or replacement of primary antibodies on sections from each tissue sample set was carried out parallel to the experimental staining. Mounted sections were allowed to stabilize for 30 min and afterwards examined with immunofluorescent and/or confocal microscopy, respectively (Leica DM2500 and Leica SP5 scanning confocal microscope; Leica, Goettingen, Germany). Image acquisition parameters were identical for each studied specimen.

### Astrocytosis and neuronal immunostaining and quantification

Quantification of lumbar ventral horn motor neurons and glial cells was performed on 4% paraformaldehyde-fixed end-stage spinal cords from MSA- and sRAGE-injected SOD1 mice. For neuronal counts, 10 μm thick sections of lumbar spinal cord were cut serially on the cryostat and stained with Cresyl Violet following standard histological protocol. For astrocytosis counts, 10 μm thick spinal cord sections were stained with primary rabbit anti-GFAP antibody (astrocyte marker; 1:150; Abcam, Cambridge, MA, USA) followed by staining with biotinylated secondary antibodies according to the standard peroxidase anti-peroxidase (PAP) protocol provided with the kit (Vectastain ABC Kit, Vector Laboratories, Burlingame, CA, USA). For each condition, 25 sections were studied, five sections per one tissue sample, 50 μm between sections within each tissue sample. Representative regions of interest, 200 × 200 μm, outlining ventral horn regions were analyzed and the same region was analyzed in each sample. All sections were examined with the Leica microscope (Leica, Wetzlar, Germany) and quantified using Image J analysis tool (Image J open source software; http://rsbweb.nih.gov/ij/).

### RNA isolation and quantitative RT-PCR

Total RNA was extracted from mouse lumbar spinal cord tissue samples using the RNeasy mini kit (Qiagen, Valencia, CA) and cDNA was synthesized with iScript cDNA Synthesis Kit (BioRad, Hercules, CA, USA). Quantitative Real Time PCR for the gene encoding mouse RAGE, Ager, was performed using the TaqMan Fast Universal Master Mix 2X with a pre-made primer set (Mm01134790_g1) (Life Technologies, Carlsbad, CA, USA). The relative expression of Ager was normalized to the expression of Ipo8 housekeeping gene. The statistical significance of differences was evaluated by Wilcoxon Signed-Rank Test.

### Lifespan, survival probability, and weight loss assessment

Survival probability is defined as the probability of surviving over a specific length of time (Goel et al., 2010). The Kaplan–Meier estimate was specifically designed to calculate the probability of survival over a specific length of time (Kaplan and Meier, 1958; Goel et al., 2010; Rich et al., 2010). In this study, we used the survival probability calculated from the onset of disease until the predicted time of death, estimated by the number of mice that survived over 130 days if no treatment was given. Kaplan–Meier survival analysis was performed using XLSTAT (Belmont, MA, USA) and the log-rank test was used to compare survival curves. The final endpoint followed by humane sacrifice was determined as 20% of initial weight loss or the animal's inability to right itself within 20 s when placed on its side. Animal's weight was monitored from the 8th week of age until the final end point as described above.

### Motor function tests

Measurement of motor function was performed using standard, widely-used and well-established procedures (NRCC, 2003). SOD1 transgenic mice begin to exhibit a decline in motor function and muscle strength at ~age 8–10 weeks, which worsens as the disease progresses. All mice were trained and acclimated to the procedure for 2 weeks prior to study start time, i.e., at the age of 8 weeks. All tests were performed twice per week until mice no longer were able to perform the tests in an appropriate and reliable manner due to the progression of motor dysfunction. In all cases, the operator was naïve to the experimental condition, and mice were given to the operator one-by-one by an investigator aware of the treatment code. Triplicate assessments were performed and the mean value was recorded for each test session. Animals were given 1-min breaks between each individual test replicate and 60 min between tests of muscle and grip strength.

#### Muscle strength test

Muscle strength was measured using the hanging wire test as described (Calvo et al., 2011). Briefly, each animal was placed on the conventional cage wire lid, turned upside down 50 cm above the padding and observed for up to 60 s. The amount of time the mouse could hold on to the wire was recorded as the latency time to falling down. As described in the Supplementary Movie 1: At the beginning of the testing period, all mice were able to hold on to the wire for the entire time (60 s). However, as the disease progressed, the time they were able to stay attached to the wire was reduced, measured as a fall latency and animals were falling off before the 60 s cut-off. More information on the procedure itself can be found in the following reference, Aartsma-Rus and van Putten (2014).

#### Grip strength

This test was used to assess muscle strength in limb muscles. Hind limb grip force was measured following the manufacturer's training guidelines (Grip strength meter, Panlab, Barcelona, Spain) by lowering the mouse toward the metal mesh grid, allowing its hind paws to attach to the grid connected to a force transducer and gently pulling backwards with consistent force by the experimenter until the grip was released. The peak force produced during the pull on the bar in grams was recorded by the transducer. Demonstration of the procedure is shown in the Supplementary Movie 2.

### Statistical analysis

All values are presented as mean ± standard error (SEM). The statistical significance of differences (*p* < 0.05) was evaluated by (GraphPad Instat, La Jolla, CA, USA) non-parametric ANOVA with Student-Newman–Keuls post-test.

## Results

### Increased expression of RAGE in ALS mouse lumbar spinal cord

**Figure 1:**
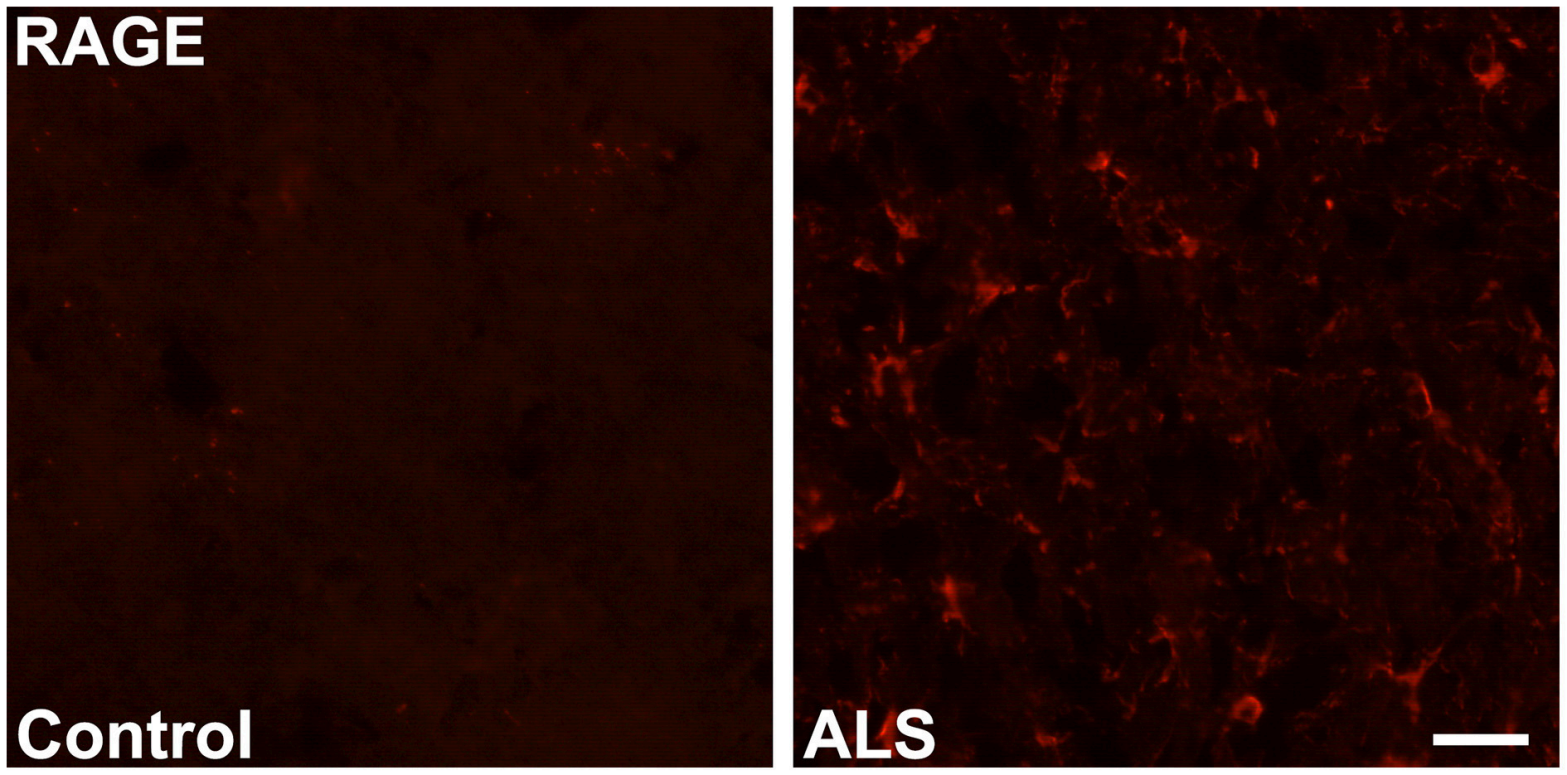
**Increased expression of RAGE in SOD1 transgenic mouse lumbar spinal cord**. Immunohistochemistry: lumbar spinal cord was stained for RAGE using standard immunofluorescent technique in control (left) and SOD1 transgenic ALS (right) mice. Representative images from *n* = 3 mice/group are shown. Scale bar = 25 μm.

**Figure 2:**
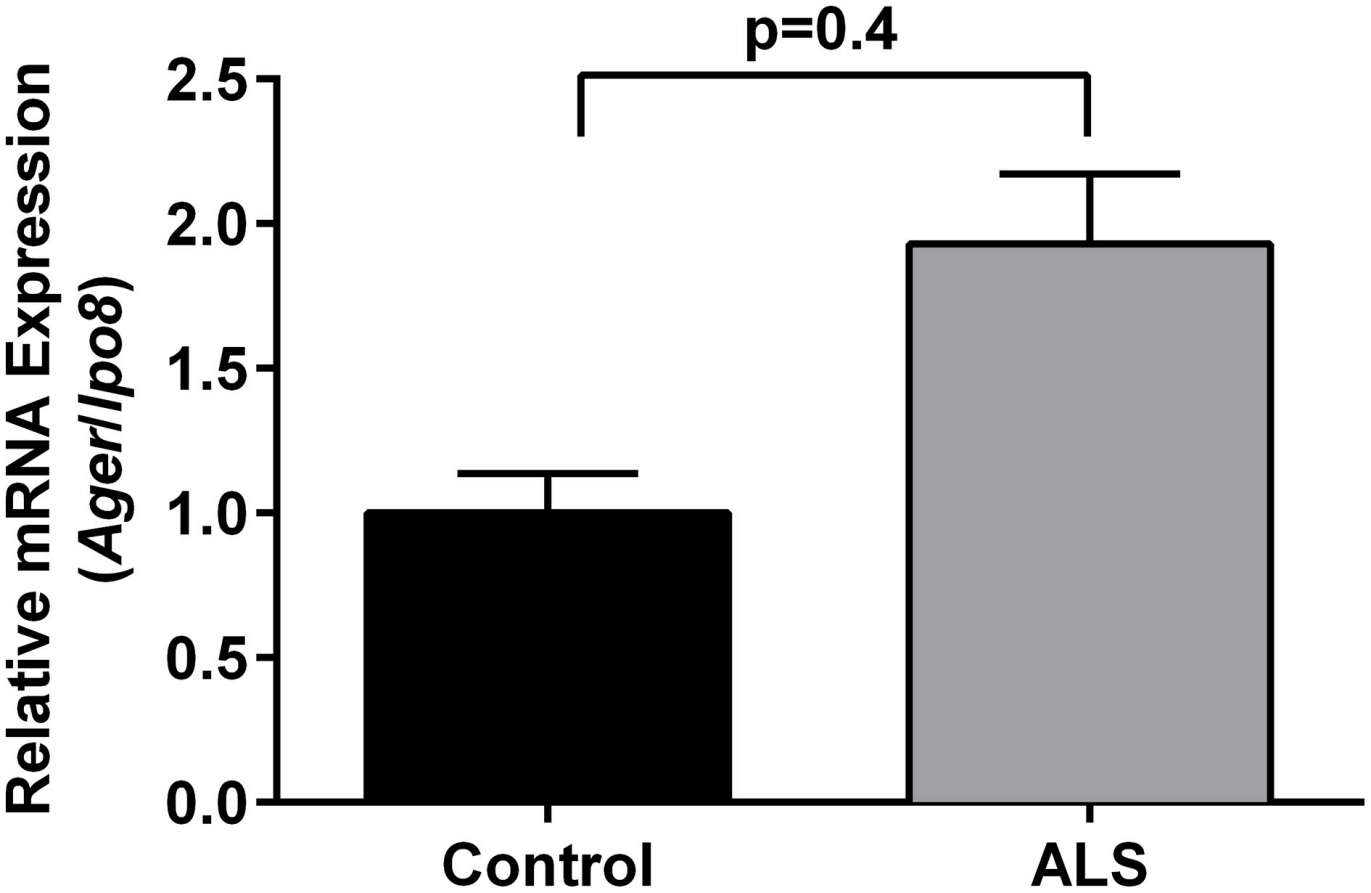
**Increased expression of RAGE in SOD1 transgenic mouse lumbar spinal cord: mRNA transcripts**. Relative mRNA expression of *Ager* mRNA transcripts was determined by quantitative RT-PCR normalized to *Ipo8* mRNA transcripts (*n* = 3 mice/group). Error bars represent SEM.

Immunohistochemistry revealed increased expression of RAGE in SOD1 transgenic vs. control mouse lumbar spinal cords at the end stage of the disease as defined above (Figure 1). Quantitative real-time PCR (qRT-PCR) of lumbar spinal cord tissue from SOD1 transgenic mice revealed trends for increased expression (2.2-fold higher) of Ager mRNA transcripts vs. controls. (Figure 2).

### Prolonged life span and improved functional performance scores in sRAGE-treated mice

**Figure 3:**
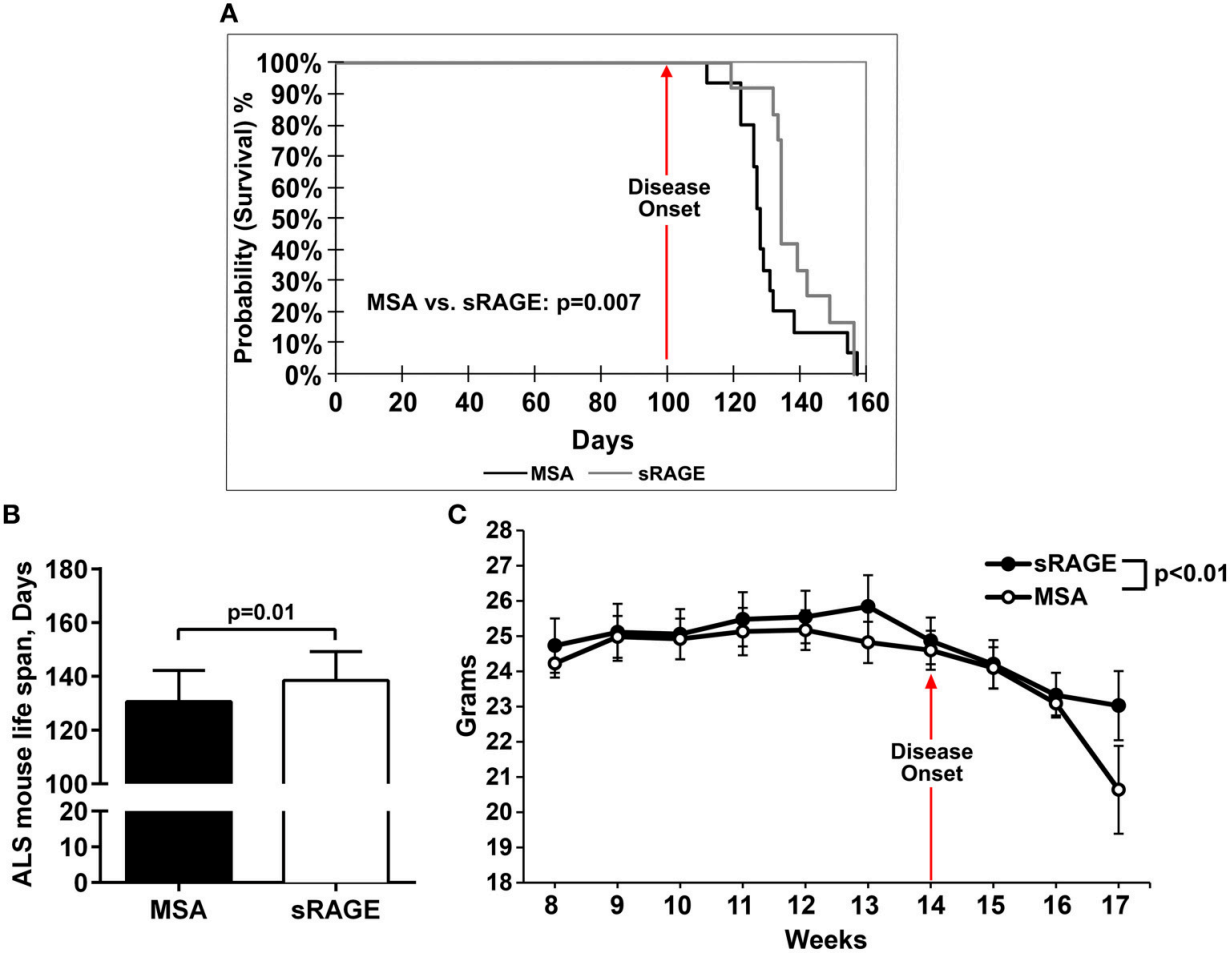
**sRAGE-treated SOD1 transgenic mice display greater longevity, higher survival probability and reduced weight loss vs. control mice**. **(A)** Kaplan–Meier estimates, i.e., percentage of surviving mice plotted vs. survival time. Black line, MSA-treated mice, gray line, sRAGE-treated mice. **(B)** Mean life span of MSA- vs. sRAGE treated mice (days). The mean number of days lived by sRAGE treated mice was significantly higher compared to MSA-treated mice. **(C)** Decrease of body weight over time for both mice groups. Error bars represent SEM; disease onset is indicated by an arrow.

To test the hypothesis that inhibition of RAGE activation would improve life span and motor performance in SOD1 transgenic mice, sRAGE, or vehicle, murine serum albumin (MSA), was administered beginning at age 8 weeks until the final stage of the disease, as defined above. Detailed analysis of the survival and longevity span revealed that male mice injected with sRAGE displayed on average a higher probability of surviving for a longer time after the disease onset; *p* = 0.007 (Figure 3A), and on average a longer lifespan compared to the MSA-treated group (138.5 ± 3.1 vs. 130.6 ± 3.1, respectively); *p* = 0.01 (Figure 3B). The onset of disease, as measured by the onset of weight decline, occurred at 14 weeks of age in control mice. sRAGE-treated mice displayed on average significantly higher body weight as compared to their MSA-treated counterparts; *p* < 0.01 (Figure 3C). In addition, the percentage of days lived by sRAGE mice was significantly higher than that of MSA mice (29.34% ± 0.66 vs. 27.67% ± 0.63, respectively (*p* = 0.02), when compared to non-transgenic SJL mice used as a reference, 100%, 472 days, as published (http://www.informatics.jax.org/external/festing/mouse/docs/SJL.shtml; Supplementary Figure 1).

**Figure 4:**
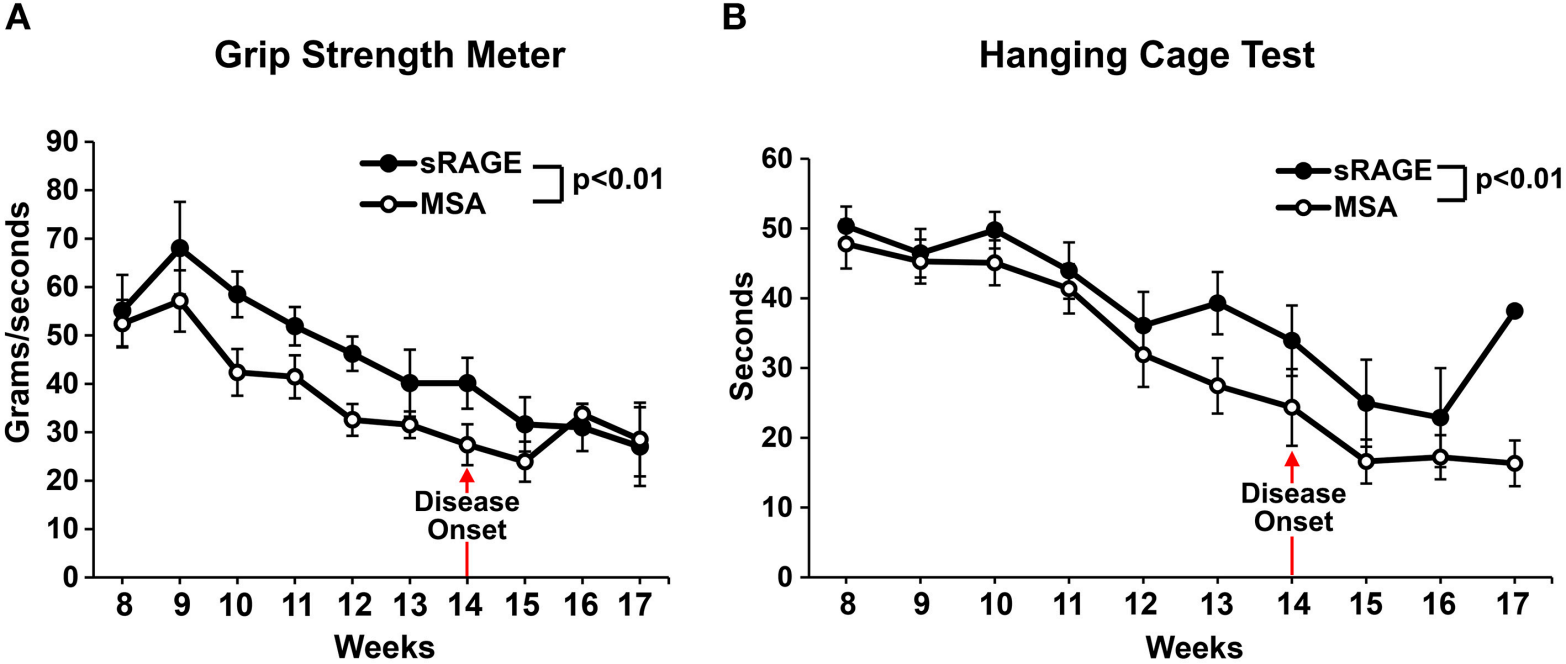
**sRAGE-treated transgenic SOD1 mice display improved motor function performance**. Results of grip strength meter test **(A)** and hanging cage test **(B)** over time for both mice groups. All tests were performed in triplicate twice a week. As the number of mice able to perform the tests was significantly reduced after week 16 and 17, hence for consistency all results are shown only until week 17; *n* = 12 mice/group. Error bars represent SEM; disease onset is indicated by an arrow.

Motor performance scores, as measured by grip strength meter test and hanging cage test (Figures 4A,B), revealed significantly higher motor performance in the sRAGE-treated mice as compared to the MSA-treated mice. Performances scores steadily declined over time and the decline was higher in the MSA-injected group, especially after the first ALS symptoms were observed (week 12–13, Figures 4A,B). Additional evidence from short videos reveals notable differences in gait, posture and movement between MSA- and sRAGE-treated animals. Specifically, at 13 and 15 weeks of age, a time point at which ALS-like symptoms were fully evident, and after ~5–7 weeks of sRAGE or vehicle treatment, SOD1 transgenic mice treated with sRAGE displayed better gait, posture, and movement (Supplementary Movies 3–5).

### Higher neuronal counts and lower astrocytosis in sRAGE-treated SOD1 mice

**Figure 5:**
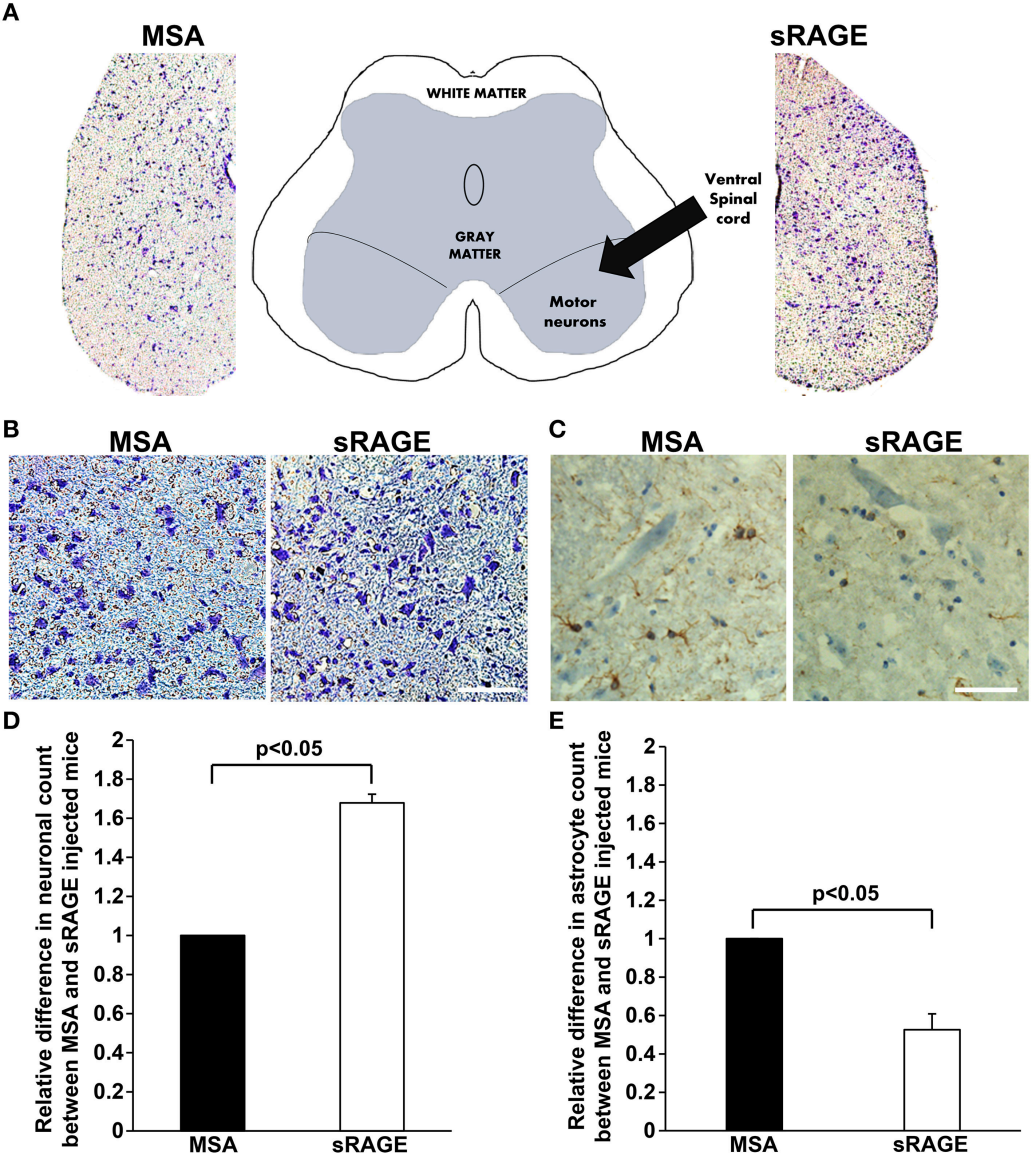
**Neuronal count and GFAP expression in terminal-stage SOD1 transgenic mouse spinal cord. (A)** A schematic diagram showing different regions of lumbar spinal cord and respective images of cresyl violet stained lumbar spinal cord regions from MSA- and sRAGE-mice as seen under the light microscope. **(B)** Cresyl violet staining revealing higher number of neurons in spinal cord of sRAGE-treated mice vs. MSA-treated mice at the terminal stage of disease. Scale bar = 75 μm. **(C)** GFAP immunostaining showing lower number of astrocytes (brown staining) in sRAGE-treated mice as compared to their MSA-treated counterparts; neurons shown here were stained with Hematoxylin and Eosin. Scale bar = 50 μm. Results of the quantitative analysis of number of neurons **(D)** and astrocytes **(E)** per region of interest in MSA- and sRAGE-treated mice. The results are presented as the relative differences between values for sRAGE and MSA group, *n* = 5 mice/group. Error bars represent SEM.

Neuronal loss and increased presence of glial cells (astrocytosis) are hallmarks of ALS pathology in the spinal cord. To determine whether sRAGE treatment affected neuronal loss or astrocytosis, we stained spinal cord samples with cresyl violet, a dye with neuronal cell affinity, and with antibodies to GFAP, a well-established astrocyte maker. We observed significant differences in the number of ventral horn motor neurons (Figures 5A,B) between MSA- and sRAGE-treated animals. sRAGE-injected mice had significantly higher number of neurons at the terminal stage of the disease, as compared to the MSA-treated counterparts (Figures 5B,D). In parallel, GFAP-immunoreactivity in the sRAGE-treated spinal cord was significantly lower than that observed in the MSA-treated spinal cord (Figures 5C,E).

## Discussion

Our study is the first report of the beneficial effects of treatment with sRAGE, which sequesters RAGE ligands and prevents their interaction with and activation of the cell surface receptor, in delaying the onset and progression of a neurodegenerative disease in the SOD1 transgenic mouse model of ALS. We observed that the onset of ALS, as measured by weight loss and performance scores, was delayed in sRAGE-treated SOD1 mice and that these mice had improved motor functional scores and prolonged life span. Additionally, we observed that these mice consistently, and for longer periods of time from the disease onset, performed better than their control treated-counterparts, manifesting overall better health condition and prolonged life span, as compared to controls. Our study also provides the first molecular evidence that, comparable to that observed in the human, expression of RAGE is increased in the SOD1 transgenic mouse lumbar spinal cord.

To date, studies on the effects of RAGE inhibition in neurodegenerative disease mouse models have been scarce and mainly limited to studies probing the effects of genetic deletion or modification of RAGE. For example, in our previous work on diabetic peripheral neuropathy, we showed that global deletion of *Ager* improves post injury sciatic nerve regeneration in type 1 diabetic mice, at least in part, by switching infiltrating macrophages from pro- to anti-inflammatory signatures, linked most likely to reduction in tissue-damaging inflammation and thereby to improvement in neuronal survival scores at the injury site (Juranek et al., 2013b). Another study in the mouse model of Parkinson's disease demonstrated that genetic deficiency of *Ager* was protective by attenuating MPTP-induced toxicity in dopaminergic neurons (Teismann et al., 2012).

Measurement of circulating levels of sRAGE in plasma/serum or the cerebrospinal fluid (CSF) of patients has been proposed to serve as a biomarker for disease onset or severity in multiple diseases (Santilli et al., 2009; Vazzana et al., 2009; Shang et al., 2010; Schmidt, 2015; Walker et al., 2015). In human studies, patients with multiple sclerosis (MS) have been found to have significantly reduced levels of sRAGE in serum (Sternberg et al., 2008) and the CSF (Glasnovic et al., 2014) when compared to controls. Circulating sRAGE levels have also been shown to be significantly lower in plasma from AD patients (Emanuele et al., 2005; Liang et al., 2013) and in serum from ALS patients (Ilzecka, 2009) vs. respective controls.

RAGE antagonists also show some promise for effective treatment in neurodegenerative diseases. Deane and colleagues identified a highly specific small molecule, FPS-ZM1, that blocks Aβ binding to the V-type extracellular domain of RAGE, thus preventing Aβ accumulation and Aβ-induced cellular toxicity in the APP (*APP*^*sw*∕*0*^) mouse model of AD (Deane et al., 2012). In a rat model of intracerebral hemorrhage (ICH), treatment with FPS-ZM1 significantly improved blood–brain barrier damage, brain edema, motor dysfunction, and nerve fiber injury (Yang et al., 2015). However, most promising progress for a RAGE antagonist as a therapeutic for a neurodegenerative disease has been in clinical trials for patients with AD (Burstein et al., 2014; Galasko et al., 2014). One study in a 400 patient cohort found that in patients with mild AD, oral administration of the RAGE antagonist TTP488 or PF-04494700 (or Azeliragon; vTv Therapeutics) demonstrated significant differences at month 18 on the ADAS-cog (Alzheimer's Disease Assessment Scale-Cognitive subscale), an established scale evaluating cognitive impairment in the assessment of AD (Burstein et al., 2014; Galasko et al., 2014). Azeliragon, produced by vTv Therapeutics, recently entered Phase 3 clinical trials.

Our previous investigation on RAGE-ligand expression in human ALS thoracic spinal cord found higher expression of RAGE and its ligands compared to controls (Juranek et al., 2015). Elevated levels of S100B, and CML-AGE, an AGE prototype, have been observed in serum of ALS patients (Sussmuth et al., 2010) and in rat motor neurons exposed to cerebrospinal fluid from ALS patients (Shobha et al., 2010). Increased S100B cerebrospinal fluid levels were also reported in Parkinson's disease (Sathe et al., 2012), Alzheimer's disease (Edwards and Robinson, 2006), and schizophrenia (Schmitt et al., 2005), implying roles for S100B in the pathogenesis of neurodegenerative diseases. Reports show that S100B triggers RAGE-mediated inflammatory responses and microglia stimulation in the brain (Bianchi et al., 2011), leading to neuronal damage and neurodegeneration and resulting in symptomatic brain disorders. In addition to potential links to inflammation, studies show that in the cerebellum of the spinocerebellar ataxia type 1 mouse model, S100B–RAGE interaction leads to increased oxidative stress and further damages neurons contributing to the progression of the disease (Hearst et al., 2011). Collectively, these studies suggest roles for the RAGE axis in the pathogenesis of neurodegenerative diseases.

Riluzole is the first and most commonly used FDA approved drug for ALS treatment. Although an earlier study suggested benefit of Riluzole in the SOD1 transgenic mouse model (Gurney et al., 1998), it was recently shown in a distinct study that Riluzole did not improve outcome of the ALS-like phenomena in these SOD1 transgenic mice (Li et al., 2013). The authors concluded used a number of different testing approaches to demonstrate that Riluzole exerted no beneficial effect on the onset and progression of the disease in these animals.

In the light of this report and the absence of any other currently approved treatment, we propose that sRAGE or other forms of RAGE blockade might fill a critical gap in this disorder as a potential single-agent or supplementary candidate for ALS treatment. As AGEs and other RAGE ligands accumulate in the ALS spinal cord, both in the human and in the murine model, we predict that RAGE contributes importantly, at least in part, to the chronic and sustained loss of neurons observed in this disorder. Further studies aimed at extending this first report are required to provide insight into molecular mechanisms of RAGE action in ALS.

## Author contributions

JJ and GD wrote the manuscript, performed and analyzed experiments. MG, RR, BK, and LK performed experiments. HL analyzed data, AMS wrote the manuscript and analyzed data.

### Conflict of interest statement

The authors declare that the research was conducted in the absence of any commercial or financial relationships that could be construed as a potential conflict of interest.

## Supplementary material

Supplementary Movie 1 **Hanging cage test: As described in the text, mice were placed on a standard cage wire lid, turned upside down and allowed to hang for 60 s**. At the beginning of the testing period, all mice were able to hold on to the wire for the entire time (60 s), but as the disease progressed, the time they were able to stay attached to the wire was reduced, measured as a fall latency and animals were falling off before the 60 s cut-off. The investigator performing the test was naïve to the experimental condition.

Supplementary Movie 2 **Grip strength meter test: As described in the text and in the manual provided by the manufacturer, the animals were allowed to grasp the metal grid and then they were pulled backwards in the horizontal plane**. The force applied to the grid before the animal lost its grip was recorded as the peak tension and measured in grams. More details on the technique available are provided here: http://www.panlab.com/en/products/grip-strength-meter. The investigator performing the test was naïve to the experimental condition.

Supplementary Movie 3 **Two male siblings, one injected with MSA (left) and the other with sRAGE (right), 14 weeks old, were placed in two separate cages**. Video emphasizes differences in gait and posture between them. While the MSA-injected mouse movement is quite reduced, the sRAGE-injected mouse is able to move more quickly and without apparent difficulty.

Supplementary Movie 4 **Same siblings, 1 week earlier (13 weeks old), already show differences in gait and coordination**. While sRAGE-injected mouse moves fast and without apparent difficulty, the MSA-injected mouse demonstrates difficulties with climbing and movement coordination.

Supplementary Movie 5 **Sibling triplets, 14–15 weeks old**. Black mouse is ALS negative and had no observable difficulty with gait or coordination. The white mouse, ALS positive, was injected with sRAGE and reveals noticeable difficulties with movement, but that these were less severe compared to the ALS positive, MSA-injected brown mouse.

Supplementary Figure 1 **The percentage of days lived by sRAGE-treated SOD1 transgenic mice was significantly higher than that of MSA-treated SOD1 transgenic mice (29.34% ± 0.66 vs. 27.67% ± 0.63, respectively; *p* = 0.02); non-transgenic SJL mice were used as the reference, 100%, 472 days)**.

## Abbreviations

AD: Alzheimer's Disease
AGEs: advanced glycation end products
ALS: Amyotrophic lateral sclerosis
CML: carboxymethyllysine
DAPI: 4′6-diamidino-2-phenylindole
EAE: experimental autoimmune encephalomyelitis
GFAP: Glial fibrillary acidic protein
HMGB1: High Mobility Group Box-1
RAGE: receptor for advanced glycation end products
MS: multiple sclerosis
MSA: murine serum albumin
sRAGE: soluble RAGE
PAP: peroxidase anti-peroxidase
PBS: phosphate buffered saline
RAGE: receptor for advanced glycation endproducts
SOD: superoxide dismutase.
