# Supplementary figures and images for: Soluble RAGE Treatment Delays Progression of Amyotrophic Lateral Sclerosis in SOD1 Mice
Source: Front Cell Neurosci. 2016 May 9;10:117. doi: 10.3389/fncel.2016.00117 (PMC4860390; doi:10.3389/fncel.2016.00117)

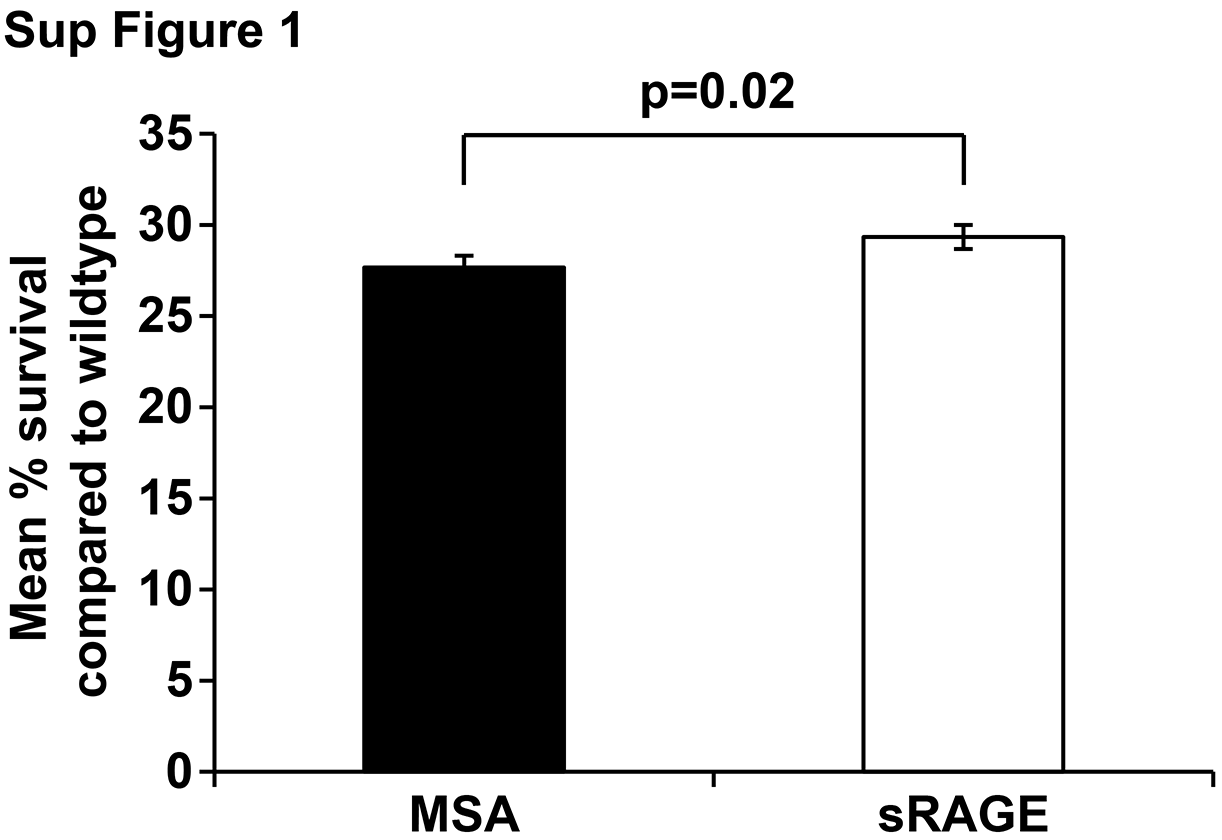

Supplement: Supplementary Figure 1 — The percentage of days lived by sRAGE-treated SOD1 transgenic mice was significantly higher than that of MSA-treated SOD1 transgenic mice (29.34% ± 0.66 vs. 27.67% ± 0.63, respectively; p = 0.02); non-transgenic SJL mice were used as the reference, 100%, 472 days). [file Image1.TIF]
